# Successful complete oral rehabilitation of a patient with osteopetrosis with extensive pre-treatments, bone grafts, dental implants and fixed bridges: a multidisciplinary case report

**DOI:** 10.1186/s12903-023-03707-3

**Published:** 2023-11-28

**Authors:** P. Kelk, A. Fasth, PLif Holgerson, M. Sjöström

**Affiliations:** 1 Department of Integrative Medical Biology, Umeå University, Umeå, Sweden; 2 Department of Pediatrics, Sahlgrenska Academy, University of Gothenburg, Gothenburg, Sweden; 3 Department of Odontology, Umeå University, Umeå, 901 85 Sweden

**Keywords:** Osteopetrosis, Hematopoietic stem cell transplantation, Iliac crest bone graft, Osseo integrated implants, Oral rehabilitation

## Abstract

**Background:**

Osteopetrosis comprises a group of inherited disorders that are rare and result in abnormal bone structure. Bone remodeling is extremely inhibited because osteoclasts are nonfunctional or lacking. This condition causes overgrowth of bone with disappearance of the bone marrow, leading to aplastic anemia; obstruction of nerve passages in the skull leads to blindness and often hearing impairment. In most cases, osteopetrosis results in oral complications such as tooth deformation, hypomineralization, and delayed or absent tooth eruption. The only curative treatment is hematopoietic stem cell transplantation (HSCT). The main treatment of the oral complications during childhood and adolescence consists in protecting the erupted teeth against caries disease through prophylactic treatment aimed at optimal oral hygiene through frequent regular dental visits throughout life. Many patients with osteopetrosis require major oral rehabilitation to treat complications of the disease. Improved results of HSCT increase the likelihood that dental professionals will encounter patients with osteopetrosis.

**Case presentation:**

In this case report, we show that individuals with osteopetrosis who have severe oral complications can be treated successfully if they are treated for osteopetrosis at an early age. The boy had his dental care in pedodontics, and regular multidisciplinary meetings were held for future treatment planning. At the age of 15, he was then referred for rehabilitation. The initial evaluations revealed no further growth in the alveolar bone. The rehabilitation was done stepwise, with extraction of malformed and malpositioned teeth. Initially, the patient received a removable partial denture followed by reconstruction of the width of the alveolar process, titanium implants, temporary fixed bridges, and finally screw-retained titanium–ceramic bridges with titanium frames for the upper and lower jaws.

**Conclusions:**

The three-year follow-up after loading indicated a stable marginal bone level and optimal oral hygiene as a result of frequent professional oral hygiene care. The patient showed no signs of symptoms from the temporomandibular joint and has adapted to the new jaw relation without any functional or phonetical issues.

## Background

Osteopetrosis (OP) comprises a group of rare hereditary bone diseases resulting in bones that are denser, heavier, and more fragile than normal, much like glass is hard but fragile [1]. Osteopetrosis was first described in 1904, by the German radiologist, Heinrich Albers-Schönberg, who discovered a benign subtype, known as autosomal dominant osteopetrosis (ADO) [2, 3].

Disease-causing variants in a number of different genes can cause osteopetrosis. The severity of the disease varies depending on the gene mutated, from mild disease accidently diagnosed at x-ray of a traumatic fracture to severe disease that is already life-threatening in a newborn baby. Most forms limit osteoclast function, while some forms affect osteoclast development. The result is an imbalance in the remodeling bone process with impaired bone resorption.

Patients with ADO, also called Albers-Schönberg disease or benign osteopetrosis, often have mild symptoms, and some individuals do not show signs or experience symptoms [4].

ADO is divided into two subtypes: ADO1 and ADO2. ADO1 is caused by an activating mutation in the *LDL5* gene resulting in increased osteoblast function [5]. ADO2 is caused by a mutation in the *CLCN7* gene, which encodes an H^+^/Cl^−^ exchange transporter protein [6] and the mutation thus impair osteoclast function. This mutation causes frequent bone fractures, and patients carry some risk of cranial nerve compression and moderate risks of anemia and immune insufficiency [4]. Patients with ADO2 are reported to have oral complications, but they are less severe compared with those in patients with autosomal recessive osteopetrosis (ARO), intermediate autosomal recessive osteopetrosis (IARO), and ADO1 [4, 7]. Reported oral complications are tooth fractures, severe caries disease, mandibular osteomyelitis, multiple dental abscesses, and mandibular fractures [8]. The prevalence of ADO varies from 2 per million in Brazil to 50 per million in Denmark [8].

Autosomal Recessive Osteopetrosis (ARO), also called malignant or infantile osteopetrosis, is the most serious type. It is often diagnosed early after birth and is lethal without treatment. The gene responsible for the most severe type, *TCIRG1,* encodes a subunit of the V-ATPase pump delivering H^+^ [9]. Bone remodeling is extremely inhibited, due to non-functional but abundant osteoclasts. This lack of osteoclast function is favorable to osteoblasts and thus tips the remodeling balance towards bone synthesis. As bone synthesis continues, pressure is exerted on the cranial nerves. Moreover, newly synthesized bone fills all the available space, which results in dense, compact bone with no bone marrow, causing anemia and immunodeficiency. Without a sufficient immune system, the patient is at high risk of severe infections [9–11]. ARO carries a significant risk of dental complications, such as caries, due to effects on enamel and dentin, as well as compressed and damaged structures of permanent teeth. Individuals with malignant OP often have little or no tooth enamel and misshaped dentin, which leads to deformed primary and permanent teeth [12]. Osteonecrosis and osteomyelitis occur more frequently in the mandible than in the maxilla, because the maxilla has vascular-rich areas and thin cortical bone, which prevent osteomyelitis and osteonecrosis [13, 14]. The estimated prevalence is between 3 and 34 individuals per million [4, 13].

IARO is similar to ARO, except IARO is typically discovered during late infancy or early childhood, and the symptoms usually develop more slowly. Variants in several genes can cause IARO [1]. In Sweden, in the Västerbotten region, there is a form of IARO caused by mutations in the *SNX10* gene. These mutations are the result of a founder effect that was traced back to the Viking age, around 950 A.D. [6, 15]. OP caused by *SNX10* mutations is more severe than other IAROs, and affected individuals develop symptoms in early infancy. The symptoms are similar to those associated with mutations in *TCIRG1*, except it is not fatal in early childhood. Palagano et al. [10] classified the Västerbotten subtype as ARO, due to its severity; however, Stattin et al. [6] argued that, because this subtype is not fatal until later childhood, it should be classified as IARO. Hematopoietic stem cell transplantation (HSCT) treatment is critical for preventing disease progression and early death [3, 6, 10].

When osteopetrosis is diagnosed in early life (i.e., before 3 years of age), HSCT can cure ARO and IARO, except when associated with mutations in some genes such as *TNFSF11*, which encodes osteoclast differentiation factor (ODF or RANKL) [16].

Independent of the type of osteopetrosis, morphological tooth deformation, hypomineralization, delayed or absent tooth eruption, and disturbed bone remodeling create conditions for caries and pulpal and periodontal infections leading to apical and marginal periodontitis, osteomyelitis, and bone necrosis. These can occur despite a successful HSCT treatment; however, HSCT increases the likelihood of tooth eruption [11, 17]. The best treatment for patients with osteopetrosis is preventive care aimed at optimal oral hygiene combined with regular dental care visits.

For the bone tissue, hyperbaric oxygen therapy increases the partial pressure of oxygen in the blood, which promotes oxygen penetration of tissues [18].

Osteopetrosis is a rare but severe disease with significant impact on the patient’s general health, life, and oral health. New and improved diagnostics and treatments have increased the likelihood that dental professionals will encounter patients with osteopetrosis. Without knowledge of appropriate treatments, dentists may even cause patients harm.

The aim of this case report is to report and share our experience of the step-by-step occlusal rehabilitation of a patient born with the Västerbotten form of osteopetrosis.

## Case presentation

**Fig. 1:**
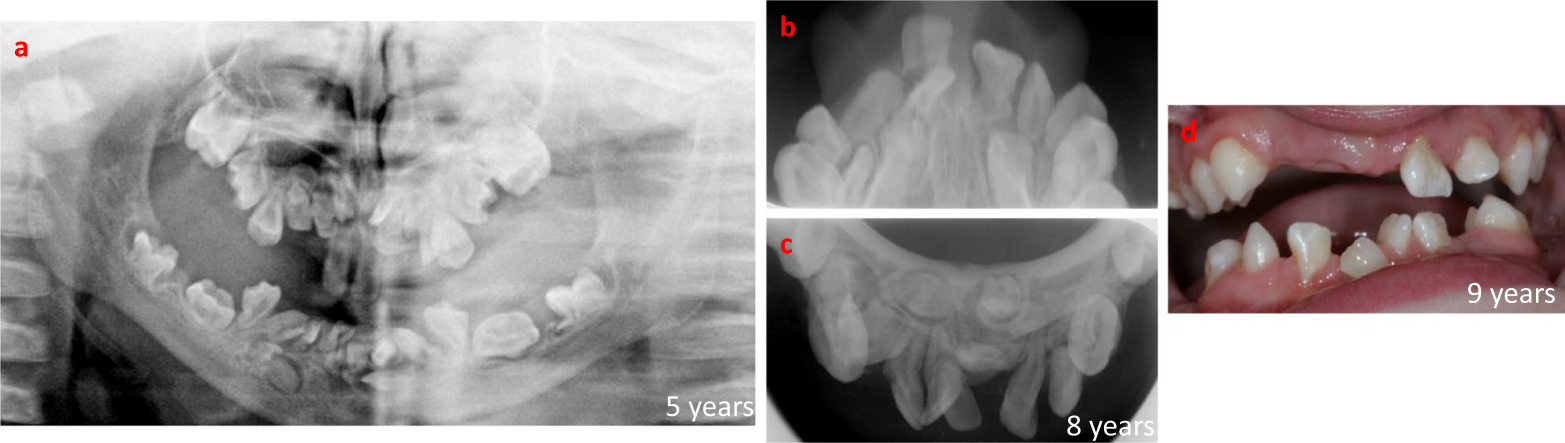
Overviews of the dentition before any dental treatment at ages 5, 8 and 9 years. **a** Panoramic radiograph at 5 years. Radiographic overview at 8 years of **b** upper jaw and **c** lower jaw. **d** Photographic overview of the dentition at 9 years

**Fig. 2:**
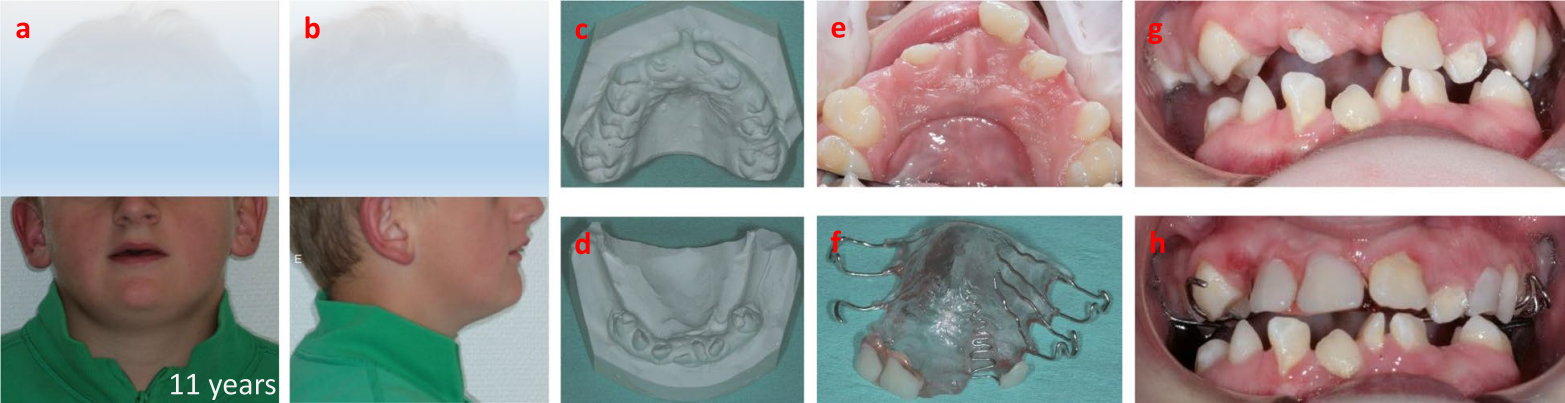
First treatment of the upper jaw with temporary removable denture at the age of 11 years. **a**, **b** Facial/extra oral photograph overviews. **c**, **d** Dental cast overviews of the dentition. **e** Photograph of the upper jaw with missing teeth that are replaced by **f** a temporary removable denture. Photographs of the dentition **g** without and **h** with temporary removable denture

**Fig. 3:**
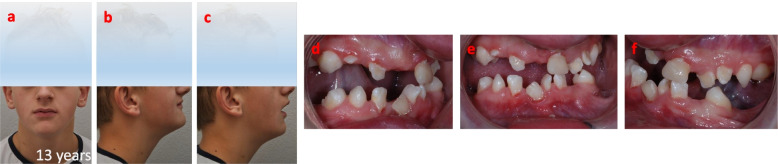
Absence of posterior occlusion at the age of 13 years that initiated treatment planning. **a**-**c** Facial/extra oral photograph overviews. **d**-**f** Photographs of the dentition and lack of posterior occlusion

**Fig. 4:**
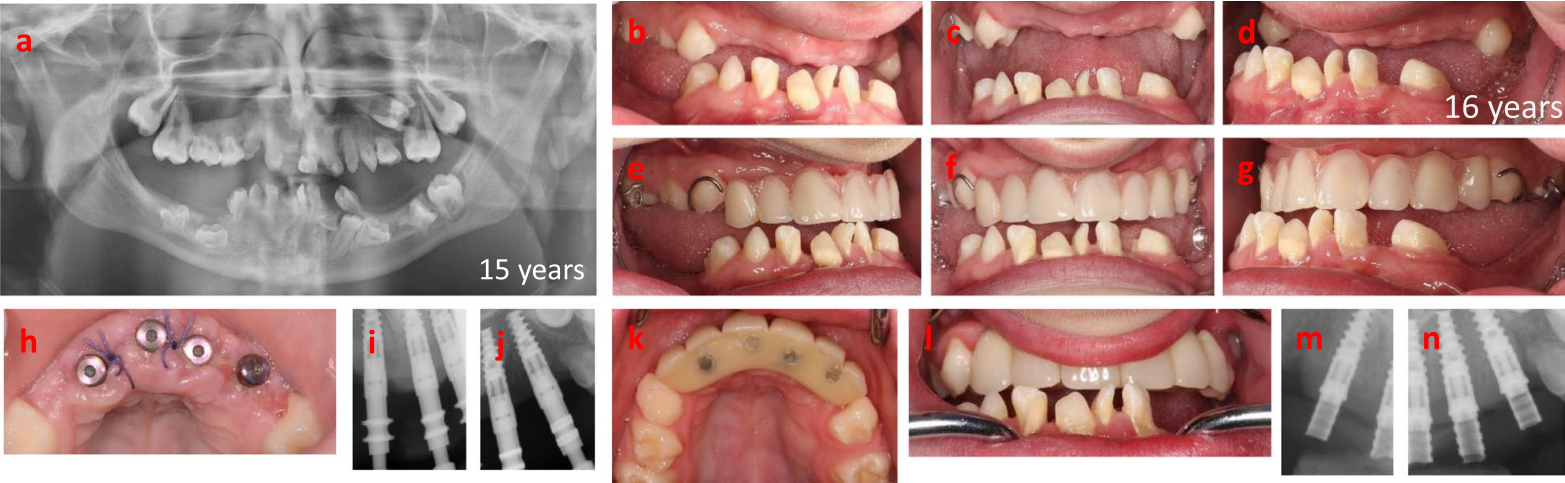
Phase 1 of the treatment at the age of 16 years. **a** Panoramic radiograph overview of the dentition at the age of 15 years, before extractions. Photographs of the dentition after extractions of teeth in positions 13–23 as shown in **b**-**d** after soft tissue healing and **e**–**g** with a new temporary removable denture. After successful bone healing and after confirmation of sufficient bone volume by CBCT, four Nobel Active, CC, NP were placed by two stage surgery in positions 12–22. **h** Occlusal photograph after the 2 nd stage surgery with healing abutments and at the time of removal for sutures. **i**, **j** Radiographs of the implants with impression copings after 6 months of healing. A temporary bridge with Polymethyl methacrylate (PMMA) was made as shown in **k** occlusal and **l** frontal photographs. **m**, **n** Radiographs of the implants + temporary bridge with temporary abutments, Nobel Biocare, CC, NP

**Fig. 5:**
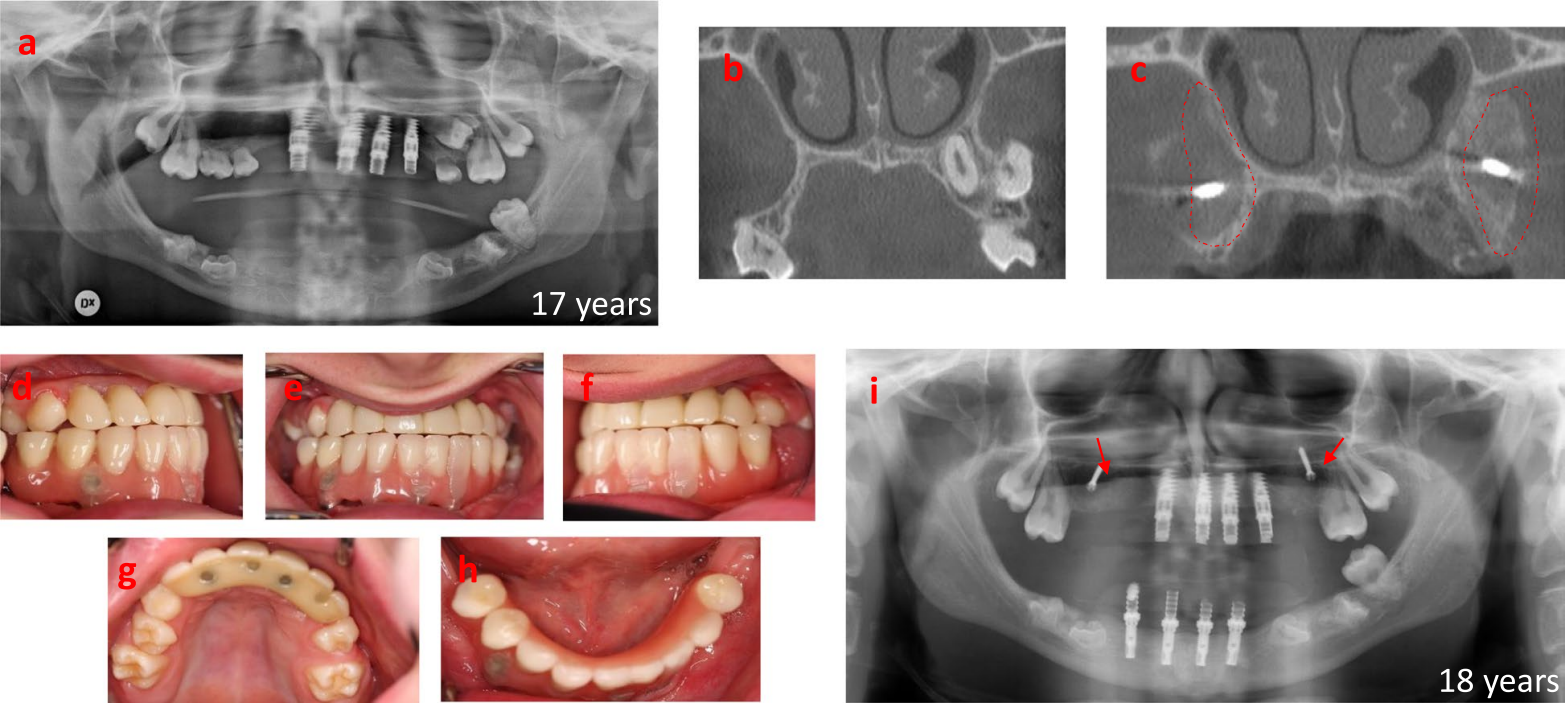
Phase 2 of the treatment at the age of 17 years. After successful healing after extraction of frontal teeth in the upper jaw and successful osseointegration of the first four implants, the teeth that were in the way for implant placement in the frontal part of the lower jaw were surgically removed. **a** Six months Post-extraction-panoramic radiograph at age of 17. Four titanium implants, Branemark System Mk III, TiUnite, RP, were placed in positions 32–42 with two stage surgery. **b** CBCT of the posterior parts of upper jaw showing a very narrow and thin bone volume. After extraction of the remaining premolars in the upper jaw and healing for three months, reconstruction of the width of the alveolar process was performed bilaterally with **c** free autologous crista iliaca block graft as highlighted in new CBCT by red lines in. **d-h** Photographs overviews of both fixed Polymethyl methacrylate (PMMA) temporary bridges, made on four implants in each jaw. **i** Radiograph panoramic overview at the age of 18 years of the fixed PMMA-bridges with temporary abutments. Notice the fixations screws of the bone grafts in the upper jaw (arrows)

A male child, born in 2001, was referred to the Department of Oral and Maxillofacial Surgery, Umeå University Hospital, in 2014. The indication for the referral was a severe malocclusion due to underdeveloped alveolar processes, partly erupted and impacted primary and permanent teeth, and severe retrognathia in the maxilla leading to a skeletal discrepancy with a pre-normal relation between the maxilla and mandible. At the age of 3 years, the patient was diagnosed with osteopetrosis due to homozygous mutations in *SNX10*. The diagnosis was delayed as he was first misdiagnosed with hereditary optic atrophy. The delayed eruption of his primary teeth and the following radiographic examination showing a dense trabecular bone structure gave the correct diagnosis. At the age of 3 years the boy underwent hematopoietic stem cell transplantation, resulting in normalized bone metabolism, which was confirmed by bone density normalization 1 year after transplantation [15]. From the age of 4 years, the boy had a normal general growth pattern except for the development of his teeth, alveolar processes, and facial bones. He was otherwise healthy without any medications or known allergies but suffered from severely impaired vision in both eyes due to his osteopetrosis. From 4 years of age, he has been followed and treated regularly by specialists in pedodontics (Fig. 1). The exfoliation of primary teeth went as normal, though the eruption of the permanent teeth was delayed. He lost his permanent incisors in the upper jaw due to trauma and was treated with a removable denture to replace teeth 12 and 11 (Fig. 2). In 2014 he was assessed for major rehabilitation of a lack of posterior occlusion (Fig. 3). The patient’s medical history explained the underdeveloped teeth, atrophic alveolar processes, and sagittal jaw discrepancy resulting in lack of posterior occlusion. During his teens he was a talented swimmer at the national level, and he is also a singer and songwriter. These physiological and social factors indicated that the boy could withstand the pretreatment phase of the rehabilitation. The boy was 13 years old at the time of referral, and the dental rehabilitation started when he was 15 years of age. The baseline for the rehabilitation is shown in Fig. 4a. Cone beam computer tomography (CBCT) revealed no signs of remaining growth in the alveolar processes. The rehabilitation started with the extraction of 8 retained and malformed teeth in the anterior maxilla (Fig. 4b-d). After 3 months of uneventful healing (with a removable partial denture, Fig. 4e-g), the bone volume of the alveolar process in the anterior maxilla was evaluated with CBCT. Four titanium implants (NobelActive NP, Nobel Biocare, Göteborg, Sweden) were placed without bone grafts in the anterior maxilla using a two-stage technique (Fig. 4 h-j). After 6 months of healing followed by abutment surgery, the patient received a fixed, screw-retained temporary bridge made of polymethyl methacrylate, in the anterior maxilla (Fig. 4k-n). The rehabilitation continued at 17 years of age with extractions of 10 impacted and malformed teeth in the mandible between the mental foramina. Once again, the healing was uneventful (Fig. 5a). All tooth extractions and implant therapy were to that point the performed under general anesthesia. Three months later a third CBCT examination was performed prior to placement of four titanium implants (Brånemark System Mk III, TiUnite, Regular platform, Gothenburg, Sweden Fig. 5i), by two-stage surgery. The healing after tooth extractions and implant placements in both maxilla and mandible have so far been uneventful. The patient received screw-retained temporary fixed bridges made of polymethyl methacrylate (Fig. 5 d-f, h) in the mandible and the maxilla (Fig. 5g). The rehabilitation continued with extraction of 3 impacted primary molars in the maxilla followed by reconstruction of the width of the alveolar process with a free autogenous crista iliaca block graft (Fig. 5b, c), with prolonged prophylaxis (phenoxymethyl penicillin 1.6 g three times daily for 8 days). Three titanium implants (NobelActive NP, Nobel Biocare, Gothenburg Sweden) were placed after 4 months of uneventful bone graft healing.

**Fig. 6:**
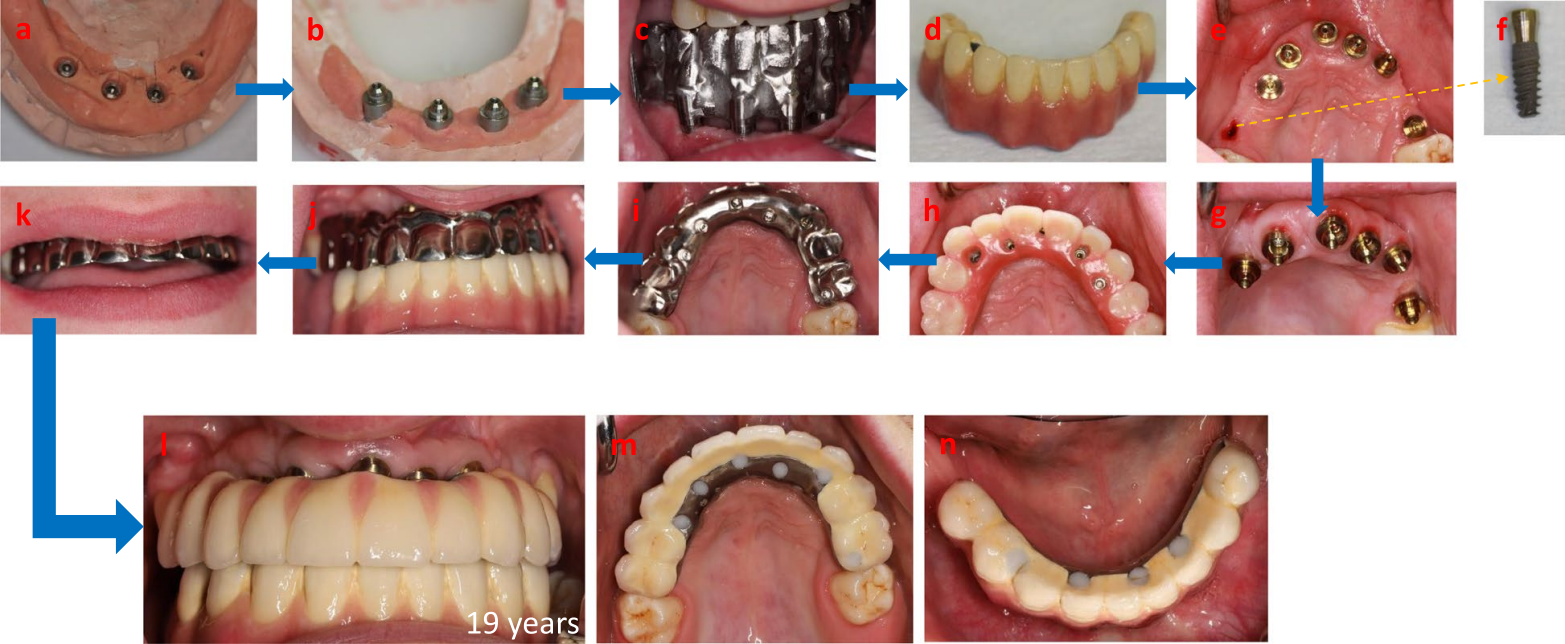
Phase 3 (final) of the treatment at the age of 19 Years. Photographs of casts of lower jaw **a** without and **b** multi-unit abutments (MUA). After impression and trial of proper dentition height, **c** the titanium frame of the fixed bridge was tested and the final **d** screw-retained titanium-ceramic bridge was made. After 6 months of bone graft healing in the upper jaw, three additional Nobel Active, CC, NP implants were placed in positions 15, 23 and 25. **e**, **f** Six months later and at the trial-stage of multi-unit abutments in the upper jaw, one of the implants (position 15) had not osseointegrated and was removed. **g** Since sufficient number of implants, total of six, had osseointegrated, the fixed bridge treatment for upper jaw could be performed anyway. **h** Vax-model trial and **i**-**k** test of occlusion of the titanium frame of the fixed bridge. **l-n** Photographs of both final screw retained titanium-ceramic bridges

**Fig. 7:**
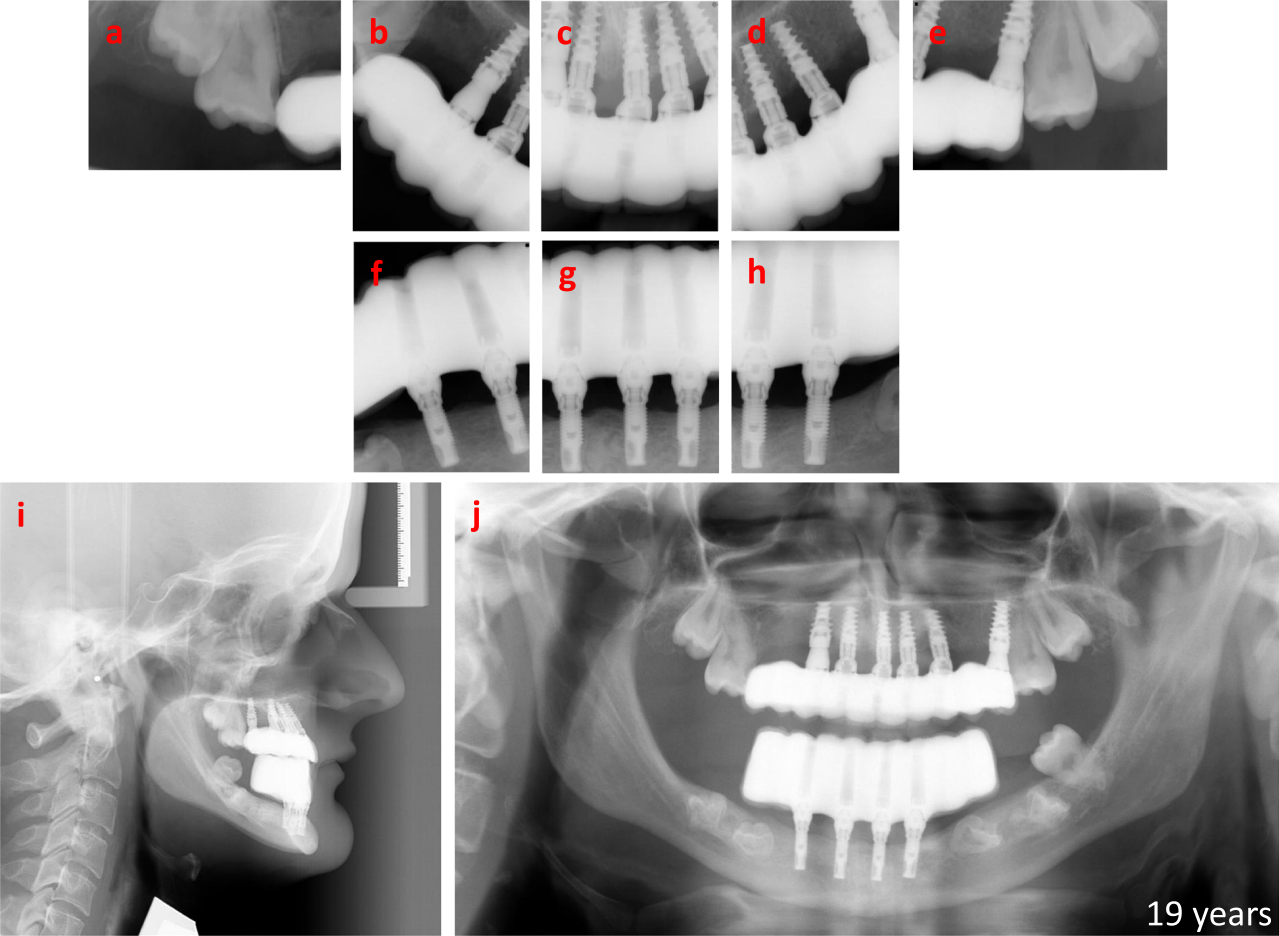
Radiographic overviews of the implant-fixed bridges at the time of initial loading at the age of 19 years. Radiographs of the screw retained-implant bridges with multi unit abutments at the stage of initial loading: **a**-**e** intraoral radiographs of the upper jaw, **f**–**h** intraoral radiographs of the lower jaw, **i** radiographic sagittal view, and **j** radiographic panoramic view

At the age of 19 years the final phase of rehabilitation took place. In both jaws, multi-unit abutments (MUA) were used to reduce the risk of prosthetic failure and to optimize oral hygiene. The mandible was rehabilitated with a screw-retained titanium–ceramic bridge with a titanium frame (Fig. 6a-d). For the maxilla, there were 7 implants placed in total (in two phases, as described earlier). At the impression stage, one implant showed signs of failed primary osseointegration and was easily removed (Fig. 6e, f). Due to the sufficient number of and evenly distributed osseointegrated implants (Fig. 6g), however, the rehabilitation of the maxilla could be performed by use of another screw-retained titanium–ceramic bridge with a titanium frame (Fig. 6h-k). The final oral rehabilitation at the loading stage is shown in Fig. 6l-n. The radiographic overviews of both implant-fixed bridges at the time of initial loading at the age of 19 years are shown in Fig. 7.

**Fig. 8:**
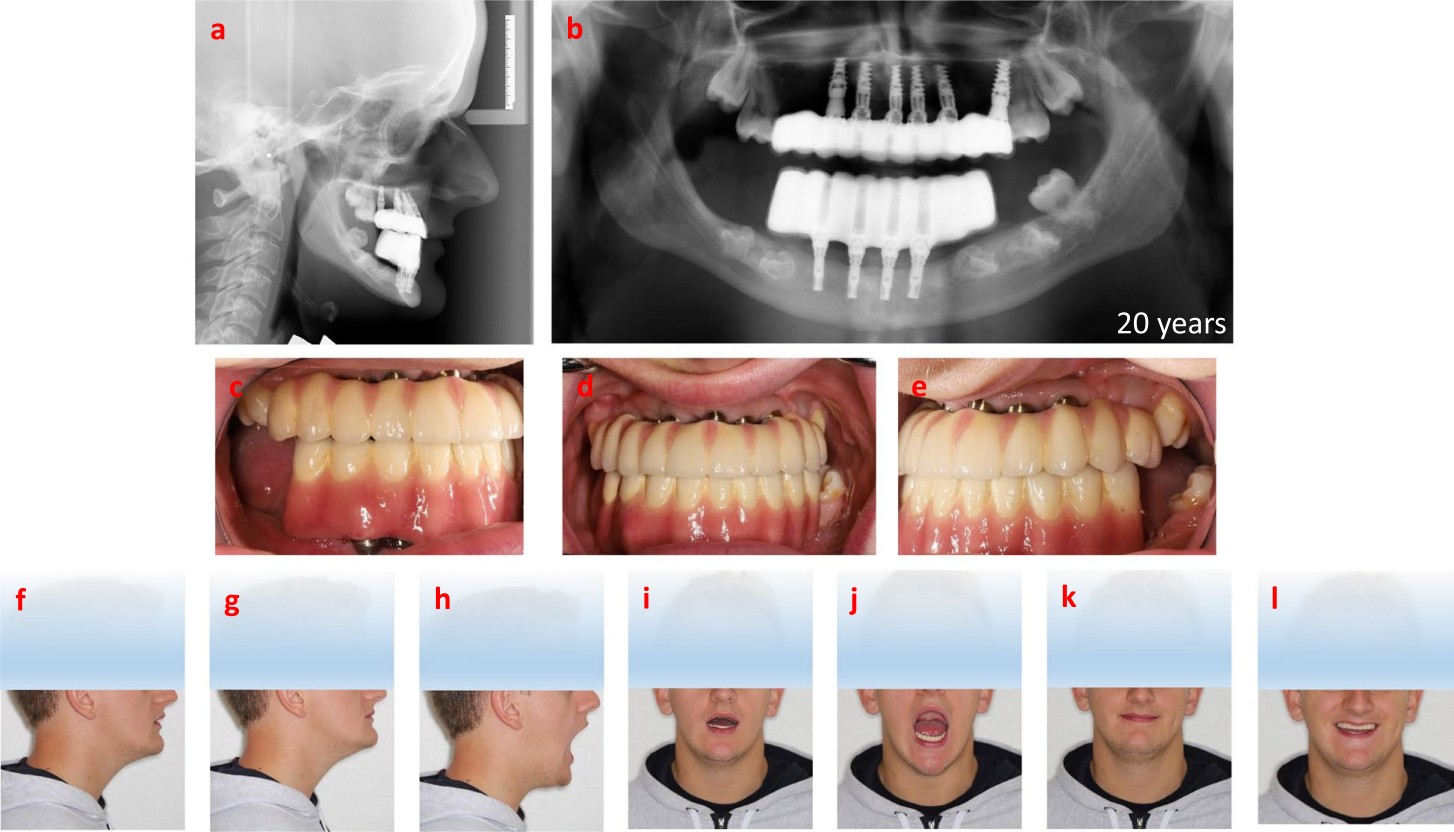
Radiographic and photographic overviews one-year post-loading of the implant-fixed bridges at the age of 20 years. **a** Radiographic sagittal view, and **b** radiographic panoramic view after one-year post-loading. **c-e** Photographs of dentition after one-year postloading. Sagittal and frontal facial photographs of **f** resting, **g** and **k** intercuspidation, **h** and **J** maximum opening, **i** small opening positions of lower Jaw in relation to upper jaw, and **l** smile line

**Fig. 9:**
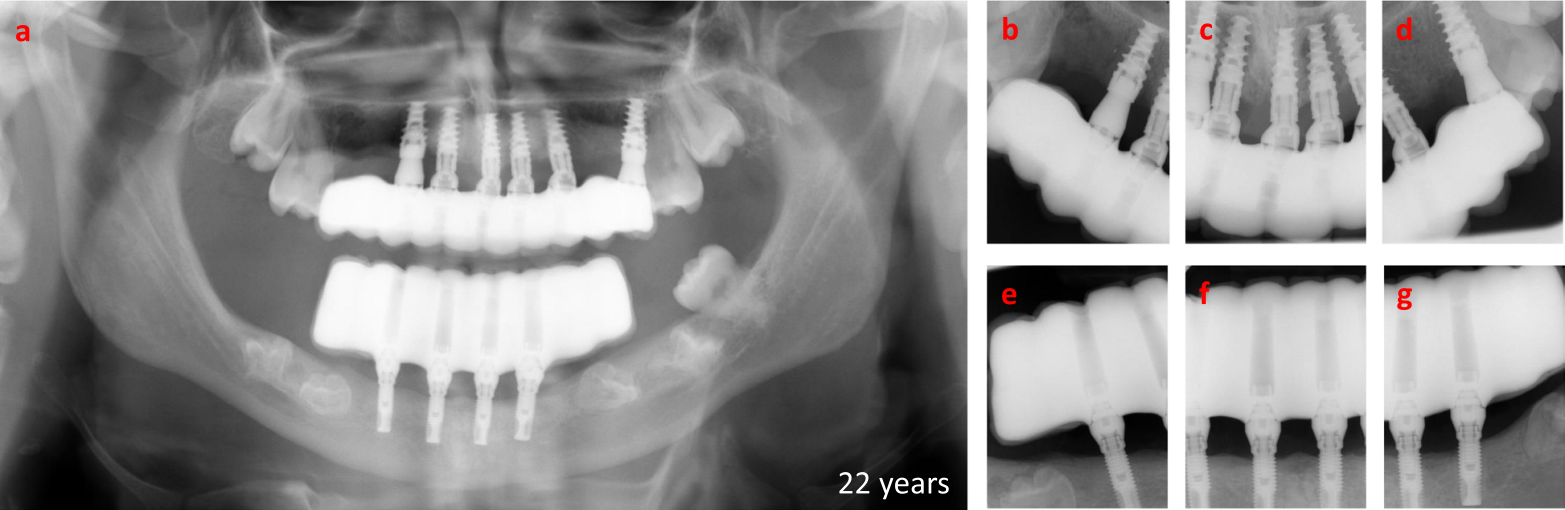
Radiographic overviews three-years post-loading of the implant-fixed bridges at the age of 22 years. **a** radiographic panoramic view, **b-d** intraoral radiographs of the upper jaw, and **e**–**g** intraoral radiographs of the lower jaw

The one-year follow up after loading indicated a stable marginal bone level (Fig. 8a, b), without any technical failures on the supra constructions (Fig. 8c-e). With regular oral hygienist visits every three months, the patient shows optimal oral hygiene. The oral rehabilitation of the patient has optimized the sagittal, and frontal relations of the mandible and maxilla, resulting in functional resting, intercuspidation relation, and movement of the jaws, with a harmonized smile line (Fig. 8f-l). Despite the substantial increase in the bite height (Fig. 8a-e), the patient has had no temporomandibular joint (TMJ) symptoms. He has adapted to the new jaw relation without any functional or phonetical issues and has in fact continued with his professional singing. The three-year follow up after loading showed an unchanged marginal bone level (Fig. 9a-g), with a minor technical failure in form av chipping of porcelain in region 21 that easily could be polished. The patient was extremely satisfied after three years regarding the esthetics and the function of the fixed implants bridges. He had marinated optimal oral hygiene by high cooperation and regular oral hygienist visits 3–4 times per year. In fact, no signs of inflammation around implants at the time of three-years follow up could be detected.

## Discussion and conclusions

This case report shows that patients with osteopetrosis with even severe oral complications can be treated if the original disease is successfully cured by HSCT. The oral rehabilitation must be performed cautiously and gradually, with meticulous evaluations between each treatment step. Although this prudence prolongs the treatment over several years, it should not disqualify the individual patient from oral rehabilitation. On the contrary, patients with osteopetrosis need oral rehabilitation and, because of the rareness of the disorder, most dental clinicians will never come in contact with these patients. The literature concerning dental rehabilitation of patients diagnosed with osteopetrosis is scarce. The use of a removable prosthesis in osteopetrosis patients has been reported with successful results [19, 20]. Ogino et al. [19] reported on a male patient whose rehabilitation consisted of surgical debridement of necrotic bone, antibiotics, and rehabilitation with removable dentures in the maxilla and mandible. In this case, the bone served as a support for the removable prosthesis. Successful treatment with a removable prosthesis is dependent on soft-tissue resistance to loading of the prosthesis. To minimize the risk for abrasions from the prosthesis, its retention must be optimal. In a case presented by Ogino et al. [19], 3 teeth in the maxilla could be saved and served to retain the prosthesis. This highlights the importance of optimal oral hygiene, which makes it possible to preserve the patient’s own dentition. Hasselby et al. performed a literature review of oral hygiene and treatment for preventing oral complications for patients with osteopetrosis. Based on the results of the literature review, they recommend continual life-long follow-ups to ensure successful treatment [21]. The titanium implant is an optimal retainer of supraconstructions. The implant must however osseointegrate into the supporting alveolar bone. Patients with osteopetrosis who have been rehabilitated with titanium implants have been reported previously [22, 23]. However, we have not found any case reconstructed with free iliac crest bone grafts followed by titanium implants supporting screw-retained bridges.

In the present case, the boy was successfully treated with HSCT [15]. During the initial pretreatment phase, we found that the bone could heal normally even after multiple tooth extractions. In addition, the patient’s history showed that a bone fracture post-HSCT had healed normally. These important findings encouraged us to continue our efforts to rehabilitate the complicated oral situation at baseline (age 13 years). Due to the original diagnosis, most of the primary and permanent teeth showed severe malformation and malposition, and no teeth had morphology or periodontal status that enabled them to be used to retain supra constructions.

Furthermore, the lack of alveolar bone and disrupted alveolar growth made it clear that treatment over a long time would be necessary to finally obtain functional and harmonized bite height and teeth relation. We approached the treatment stepwise, evaluating the healing process after teeth extractions, reconstructions, and implant placements. Each step was carefully assessed, and the outcome was discussed within the reconstruction team as well as with the patient and his parents before the next step was taken. In addition, we used conservative surgical techniques, for instance two-stage surgery during implant placement and prolonged healing periods (after bone reconstruction and implant placement). We also enabled oral hygiene conditions with optimally designed supra constructions and the use of abutments for easy future repair of eventual technical failures.

Other important factors for the long-term success of the treatment were frequent professional oral hygiene maintenance, sustained patient co-operation, and annual prosthetic follow-ups with re-examination of marginal bone height and checking for signs of inflammation. The results from the three-year follow-up support this strategy. We recommend that oral rehabilitation of patients with osteopetrosis or conditions with persistent sequelae from the osteopetrosis diagnosis should be planned and treated by a team that includes several odontological specialties.

## Data Availability

All data and materials included in this case presentation are based on the patient’s medical record. No person except the patient and the responsible doctor has a right to the patient’s medical record.

## Abbreviations

OP: Osteopetrosis
HSCT: Hematopoietic stem cell transplantation
ADO: Autosomal dominant osteopetrosis
ARO: Autosomal Recessive Osteopetrosis
IARO: Intermediate Autosomal Recessive Osteopetrosis
